# Contributing Factors to the Rise in Adolescent Anxiety and Associated Mental Health Disorders: A Narrative Review of Current Literature

**DOI:** 10.1111/jcap.70009

**Published:** 2024-12-30

**Authors:** Thea L. Anderson, Rasa Valiauga, Christian Tallo, Catriona Blythe Hong, Shaminy Manoranjithan, Catherine Domingo, Manasvi Paudel, Ana Untaroiu, Samantha Barr, Kate Goldhaber

**Affiliations:** ^1^ University of Connecticut School of Medicine Farmington Connecticut USA; ^2^ Stritch School of Medicine Loyola University Chicago Maywood Illinois USA; ^3^ University of Missouri School of Medicine Columbia Missouri USA; ^4^ Northeast Ohio Medical University Rootstown Ohio USA; ^5^ Rosalind Franklin University of Medicine and Science North Chicago Illinois USA; ^6^ Medical College of Wisconsin Milwaukee Wisconsin USA; ^7^ University of Wisconsin School of Medicine and Public Health Madison Wisconsin USA

**Keywords:** adolescence, anxiety, mental health

## Abstract

**Background:**

The prevalence of anxiety among adolescents has seen a notable increase in recent years, becoming a significant public health concern. In fact, anxiety is substantially more prevalent in Generation Z (individuals born between 1997 and 2012) than in any of the past three generations. We aimed to examine what factors contribute to the increased prevalence in teen anxiety and identify points of intervention.

**Methods:**

This study employed a narrative review method. We performed a literature search of the PubMed, ScienceDirect, and Medline databases and identified original research and review articles discussing increased anxiety and other mental health disorders in Generation Z.

**Results:**

We provide a comprehensive overview of the factors contributing to the increased rates of adolescent anxiety, including academic pressures, social media influence, family dynamics, and broader societal stressors.

**Conclusions:**

In this narrative review, we examine the multifaceted nature of adolescent anxiety, identifying contributing factors. Additionally, we discuss potential clinical, educational, and community‐based interventions to prevent and treat adolescent anxiety. By understanding and addressing the underlying causes of anxiety, it is possible to mitigate its impact and promote healthier developmental trajectories for young individuals.

## 1 Introduction

Adolescent anxiety and associated mental health disorders have emerged as a pressing concern in recent years, with a growing body of evidence suggesting a significant increase in prevalence rates. The COVID‐19 pandemic has resulted in considerable research regarding the impact of the pandemic and resulting social isolation on adolescent mental health. A systematic review of 61 articles found significant increases in the rates of anxiety and depression among children and adolescents worldwide compared to pre‐pandemic levels (Panchal et al. 2023). Key risk factors identified by the studies included in the review were mental health problems before the pandemic and excessive media exposure, while strong family communication and social support were protective against the development of mental illness. Additionally, several studies found that individuals with neurodevelopmental disorders and special educational needs exhibited more emotional problems than neurotypical peers (Nonweiler et al. 2020; Waite et al. 2021). Pre‐existing disparities were further exacerbated by the pandemic, disproportionately impacting youth from minority backgrounds (Fortuna et al. 2023). A study evaluating changes in the rates of depression, anxiety, and suicide risk in youths aged 8–20 years and spanning 2015–2022 found that the greatest increase in depression and anxiety was among Hispanic and Asian females, while the largest suicide risk was observed in Asian females and Black females (Prichett et al. 2024). Recent industry reports emphasize comparable trends in the Gen Z population.

The Gallup–Walton Family Foundation Voices of Gen Z Report, based on a survey conducted April–May 2023, highlights the alarming state of mental health among young people (Walton Family Foundation 2024). The report found that only 47% of Gen Z members (aged 12–26) consider themselves to be thriving, compared to 59% of millennials, 57% of Gen X, and 52% of baby boomers. Part of this staggering number may be explained by the finding that Gen Z is more likely to report concerns regarding mental health (Bethune 2019). However, mental health is multifactorial, and these rising numbers can also be attributed to various cultural influences, including biological factors, social media, nuclear family dynamics, academic pressures, extracurriculars, and global uncertainty (McKinsey & Company 2024).

The toll of living with a mental illness is severe, and detrimental coping methods may exacerbate the challenges faced by the individual. According to a study examining changes in medication usage between January 2016 and December 2022, the monthly antidepressant dispensing rate for children, adolescents, and young adults aged 12–25 years increased by 66.3% in the defined time period (Chua et al. 2024). While these medications can be extremely effective in managing anxiety and depression, they may provoke notable side effects as antidepressants carry a Federal Drug Administration black box warning regarding the potential to increase the risk of suicidality in those younger than 25 years old (Reeves and Ladner 2010). Roughly 30% of adolescents will experience an anxiety‐related disorder, a statistic that is continuing to rise, and the rate of hospital admissions for suicidal teenagers has doubled over the past decade (Chiu, Falk, and Walkup 2016; Plemmons et al. 2018). This reflects the importance of identifying and understanding external and internal contributors to mental health and promoting healthy coping mechanisms.

The growing prevalence of anxiety in adolescents is paralleled by the rising demands placed on mental health professionals, highlighting a critical gap in mental health services. One meta‐analysis found that the combined treatment rate for any mental health disorder among children and adolescents was only 38%, underscoring the need for more accessible and comprehensive care (Wang et al. 2023). Psychiatric nurse practitioners play a pivotal role in diagnosing, treating, and prescribing for patients in both inpatient and outpatient settings (Muench and Fraze 2022). Given the severe shortages of child and adolescent psychiatrists, psychiatric nurse practitioners can bridge the gap in access to mental health services, providing crucial support to this vulnerable population (Yang, Idzik, and Evans 2021).

The rise in adolescent anxiety has far‐reaching implications not only for individual well‐being but also for family dynamics and societal functioning. Understanding the underlying elements associated with this phenomenon is essential for developing effective prevention and intervention strategies. This narrative review synthesizes current literature on the factors contributing to the rise in adolescent anxiety and related mental health disorders. By examining a wide range of potential influences—individual, familial, societal, and environmental—we aim to provide a comprehensive overview of the complex interplay affecting adolescent mental health. Through this examination, we hope to offer insights that empower mental health professionals to better address the growing demands of adolescent care. Our review aims to equip psychiatric nurse practitioners with the necessary knowledge to deliver more tailored and effective care.

## 2 Methods

The present study employed a narrative review. We searched for research articles using the PubMed, ScienceDirect, and Medline databases. Key search terms included the following: (adolescent anxiety OR anxiety OR mental health) AND (genetics OR environment OR technology OR social media OR family OR academic pressure OR academic stress OR extracurricular activity OR politics OR climate change AND adolescent anxiety). Our inclusion criteria were as follows: (1) articles that are written in English and published 2000–2023, (2) discuss mental health problems experienced by adolescents and young adults aged 10–21 (middle school through college), and (3) full article texts are available. Any articles not meeting all three criteria were excluded from the review. This search yielded 56 peer‐reviewed articles, which are synthesized and presented in this manuscript. We also included recent information from polling organizations in the United States to provide data representative of the broader population.

### 2.1 Biological Factors

The etiology of anxiety disorders is a complex interplay between genetic predisposition and environmental factors, with emerging research investigating the nuances of this relationship. The 30%–40% heritability of anxiety disorders underscores the significance of genetic contributions (Hettema, Neale, and Kendler 2001). Evidence from family and twin studies highlights a four‐ to six‐fold increased risk in first‐degree relatives. However, the polygenic nature of anxiety makes pinpointing specific genes challenging. Recent genome‐wide association studies identified the PDE4B gene as a robust risk locus associated with anxiety and stress‐related disorders, along with correlation to other psychiatric traits, including depression, neuroticism, and schizophrenia (Meier and Deckert 2019; Meier et al. 2019).

In tandem with genetic factors, environmental elements, notably pollution and toxins, have garnered attention in recent years. Common pollutants include particulate matter and nitrogen dioxide. Increasing exposure to environmental toxins, including endocrine‐disrupting chemicals such as bisphenol A and phthalate, has been an avenue of investigation as they have been shown to affect pre‐ and post‐natal development and behavior (Bakoyiannis, Kitraki, and Stamatakis 2021). Moreover, research suggests air pollution plays a role in the development of anxiety and depressive disorders, with approximately 73% of studies indicating a positive correlation (Zundel et al. 2022). This significant correlation underscores the effect of pollution on the brain, including increased inflammation, oxidative stress, and neurotransmitter imbalance.

The relationship between genetic predisposition and environmental influences is further illuminated by epigenetic considerations, where DNA hypermethylation in neurodevelopment pathways has been identified in children and adolescents with anxiety. Additionally, a 2021 study highlighted autonomic hypersensitivity to adrenergic stimulation and dysregulation of ventromedial prefrontal cortex activity in individuals with generalized anxiety disorder (Teed et al. 2022). Overall, these insights collectively show potential treatment targets while emphasizing further need for a comprehensive understanding of the neurobiological and genetic underpinnings of anxiety.

### 2.2 Digital Technology and Social Media

The impact of social media on adolescent mental health is a complex issue that has garnered significant attention from researchers, educators, and policymakers. The emergence of social media platforms has revolutionized the way adolescents interact with peers and perceive themselves, bringing both positive and negative implications for mental health. A 2023 Pew Research Center survey found that 95% of teens ages 13–17 have smartphones and 96% are on the internet daily. Additionally, 1 in 5 teens report using YouTube and TikTok almost constantly (Auxier 2020). The prevalence of smartphones allows teenagers constant access to information and facilitates multitasking in their daily activities. However, the increased usage of digital technology raises concerns about potential negative effects on mental health, such as screen time‐related issues and exposure to negative online content (Orben 2020).

Educational pursuits are also influenced by digital tools. In the United States, 6 in 10 eighth‐graders rely on computers and the internet for research, online learning, and collaborative projects daily (Auxier 2020). The addictive potential of social media, characterized by compulsive use and a preoccupation with online interactions, has been linked to detrimental mental health outcomes (Shannon et al. 2022). Yet, this relationship is complex, influenced by the quality of online interactions and offline social support networks. The distinction between active and passive use of social media further complicates this picture, with active engagement (posting, commenting, etc.) potentially offering protective benefits against loneliness and depression, in contrast to passive consumption such as scrolling through feeds (Vaingankar et al. 2022).

Therefore, it is crucial to acknowledge the positive dimensions of social media, particularly in terms of mental health awareness and online support. The anonymity and accessibility of online platforms can facilitate the formation of supportive communities, offering a lifeline to adolescents who may not have a robust support network in their offline lives (Naslund et al. 2020). Studies provide evidence for the positive impact of social media on reducing symptoms of depression and increasing mental health literacy among adolescents (Hassen et al. 2022). These findings underscore the potential for social media to serve as a valuable tool in promoting mental well‐being, if users engage in healthy, supportive online behaviors.

The impact of social media on adolescent mental health is profoundly shaped by the interplay of individual vulnerabilities, the nature of social media engagement, and the broader social context (Fassi et al. 2024). This emphasizes the importance of adopting a multidimensional approach to understanding and addressing the mental health implications of social media use. Future research and interventions should not only aim to mitigate the risks associated with social media use but also leverage its potential as a resource for promoting mental health and resilience among adolescents. This balanced approach necessitates a collaborative effort among researchers, educators, and policymakers to aid mental health professionals in developing evidence‐based strategies that empower adolescents to navigate the digital landscape in ways that promote their mental health and well‐being.

### 2.3 Nuclear Family Dynamics

Research has documented shifts in family structure and its potential implications for child and adolescent mental health outcomes (An et al. 2024). One significant change has been a decline in “alloparenting” practices, which involves the care and support of children by individuals other than their biological parents, such as grandparents, aunts, uncles, and community members. Extended family support has been a widespread practice in many cultures throughout history, and studies suggest it can provide additional resources for both parents and children (Herlosky and Crittenden 2021; Kenkel, Perkeybile, and Carter 2017). This shared responsibility for child‐rearing can provide a buffer against the stresses and demands of parenting, allowing for a more distributed workload and increased emotional and practical support.

However, as societies have become more individualistic and geographically dispersed, the role of extended family and community in child‐rearing has diminished (Itao and Kaneko 2021). This shift has placed a greater burden on parents, particularly those in single‐parent or dual‐working households, leading to increased strain on family relationships. The challenges faced by single parents, such as financial stress, social stigma, and limited access to support networks, can be further exacerbated by the absence of alloparental support (Taylor and Conger 2017). This combination of factors can contribute to elevated rates of parental psychopathology and negative parenting behaviors, which in turn increase the risk of anxiety and other mental health problems in children (Daryanani et al. 2016; Grüning Parache et al. 2023; Maitri and Venus 2022).

The decline in alloparenting practices can be partially attributed to increased geographic mobility, as individuals and families are more likely to live farther away from their extended kin networks (Sadruddin et al. 2019). While this mobility can provide access to new opportunities and resources, it can also lead to increased isolation and a lack of social support. The absence of alloparental support may also have direct effects on children's emotional development. A 2020 study found that children who received consistent care and support from extended family members had lower levels of anxiety and depression compared to those who did not (Van IJzendoorn et al. 2020). This suggests that the presence of multiple, supportive caregivers can provide children with a sense of security and emotional resilience, which may help protect against the development of anxiety disorders.

As modern societies continue to grapple with the challenges of balancing work and family life, recognizing the importance of extended family support and finding ways to foster supportive, extended family and community networks may be crucial in promoting the mental well‐being of both parents and children. This is particularly important for single‐parent families, who may face additional barriers to accessing support. Future research should investigate the specific mechanisms by which family structure impacts adolescent anxiety and the development of evidence‐based interventions that account for diverse family configurations.

### 2.4 Academic Pressure

As the pursuit of higher education continues to be tied to career opportunities and financial success, and with increasing competition to gain admission into top universities for undergraduate and graduate programs, students are under more pressure than ever before to excel academically. Academic demands have undoubtedly contributed to the growing number of teenagers experiencing anxiety and other mental health issues, both in the United States and abroad (Pascoe, Hetrick, and Parker 2020). A recently conducted systematic review of studies across the world examining academic pressure and adolescent mental health found that 48 out of the 52 studies showed a positive correlation between academic pressure and poor adolescent mental health outcomes (Steare et al. 2023). Additionally, a small (*n* = 399) cross‐sectional study of Greek adolescents aged 12–18 years found that older age, female sex, single‐parent household, parental profession, and number of hours spent studying were positively correlated with stress and anxiety (Moustaka et al. 2023).

Similarly, two longitudinal studies conducted on adolescents in China and India both showed that students with heavy academic pressure were more likely to suffer from anxiety disorders (Hua et al. 2023; Trevethan et al. 2022). In the US, Franciscan University in Ohio sought an explanation for the drastic increase in student visits to the campus counseling center and found that academic performance and the pressure to succeed were the top concerns amongst the 374 undergraduates surveyed, suggesting academic pressure may be partially to blame for increased mental health issues amongst the students (Beiter et al. 2015). Furthermore, a study investigating the mental health impact of Early Entrance to College Programs found that the additional academic pressure in these accelerated programs increased the students’ susceptibility to developing mental health disorders, including anxiety and depression (Singh et al. 2021).

The aforementioned studies all suggest that high academic demands are having negative impacts on youth mental health, contributing to the increased prevalence of anxiety amongst teens cross‐culturally. Further research using larger cohorts is needed to delineate causality and understand where interventions may be appropriate to encourage academic excellence while minimizing undue stress.

### 2.5 Extracurricular Activities

In addition to classroom performance, engagement in extracurricular activities is often considered a vital component of academic success. From one standpoint, intensive extracurriculars can lead to “overscheduling” and increased feelings of anxiety, as demonstrated in a 2007 study evaluating time spent on extracurricular activities in high school students in New York. While anxiety was reported at higher levels in students with more time spent on extracurriculars, similar results were not observed for depression or somatization (Melman, Little, and Akin‐Little 2007). However, most research suggests that engagement in extracurricular activities offers a protective effect on adolescent mental health. One study examining 332 adolescents at routine medical appointments assessed the role of multiple social factors on the prevalence of depressive symptoms, and found that those with more extracurricular activities (clubs, organized sports teams etc.) and high equality family relationships had significantly lower depressive symptoms (Mason et al. 2009). Similarly, a study of senior high school students in Greece found that those who engaged in >11 hours per week of extracurricular activity reported lower levels of anxiety, whereas more time spent on school‐related activities was associated with higher levels of anxiety (Lazaratou et al. 2013). A longitudinal study investigating the role of after‐school activities on developmental outcomes found that sports participation was associated with lower anxiety and depressive symptoms (Fauth, Roth, and Brooks‐Gunn 2007). However, multiple studies note the importance of examining the varying types of extracurricular activities, as findings thus far reveal varying implications on anxiety, depression, stress, resiliency, substance use, and delinquency (Farb and Matjasko 2012).

Interestingly, a recent study examining screen time versus participation in extracurricular activities in Canadian seventh‐graders found that adolescents who engaged in extracurricular activities reported significantly less recreational screen time, and that this was associated with better mental health in both boys and girls (Oberle et al. 2020). Conversely, two or more hours of screen use after school was linked to poorer mental health, especially for girls, suggesting that active engagement in extracurricular activities may fill the free time that may have otherwise been used for more isolating and/or detrimental activities such as browsing on social media.

It is well established that certain extracurriculars, specifically organized sports, can have a protective effect on anxiety and depressive symptoms and foster resilience (Binsinger, Laure, and Ambard 2006; Panza et al. 2020; Ruvalcaba et al. 2017). A meta‐analysis reviewing 29 articles and with a cumulative sample size of 122,056 participants found a small but significant effect between adolescents involved in sports and lower rates of anxiety and depression; however, a causal effect could not be established (Panza et al. 2020). Similarly, a 2006 study from France found that regular extracurricular sports practice is associated with better levels of self‐esteem and trait anxiety among young adolescents.

Gender differences emerged in the protective effects of extracurricular exercise. Extracurricular exercise in girls was more protective against larger fluctuations in Rosenberg's self‐esteem scores, while boys demonstrated no significant protection against either moderate or large fluctuations in self‐esteem score or trait anxiety as measured by Spielberger's anxiety scale (Binsinger, Laure, and Ambard 2006). Taken together, these findings suggest extracurricular involvement is largely protective for adolescents’ mental health, yet further research is needed to establish causation and characterize specifics of engagement type and intensity of time spent. There is also a need for further investigation of the relationship between intensive involvement in extracurriculars for the purposes of academic success versus participation for the sake of leisure or enjoyment and respective impacts on adolescent anxiety.

### 2.6 Political and Environmental Uncertainty

Intertwined political and environmental policies exacerbate stress, prompting scrutiny of these factors in adolescent anxiety. In one study conducted on over 10,000 young adults aged 16–25 from 10 countries, most participants were either extremely or moderately worried about climate change, and this anxiety was correlated with perceived inadequate government response or feelings of betrayal (Hickman et al. 2021). Youth in poorer countries facing a greater impact from climate change were more likely to report higher frequencies of worrying and feel that this impacts their daily lives. The long‐term implications of climate change, such as displacement, food insecurity, and ecosystem collapse, can contribute to a sense of hopelessness and despair among adolescents (Cianconi, Betrò, and Janiri 2020). This anxiety associated with environmental uncertainty can also negatively impact their academic engagement and prosocial behavior, factors that strongly influence an adolescent's future (Kong and Zeng 2023).

Various factors, such as environmental policy, elections, and safety concerns, can lead to political uncertainty. One study found that over 50% of children have anxiety about at least one political issue, with the environment and gun violence being the two with highest concerns, while another study showed that 54% of those who used social media during a recent election identified it as a significant source of their stress (Caporino, Exley, and Latzman 2020; Dejonckheere, Fisher, and Chang 2018). Along with political instability, social unrest has been identified as a co‐contributor to adolescent anxiety. Exposure to political violence, either directly or through media, has increased rates of posttraumatic stress disorder, depression, and anxiety among youth (Slone and Mann 2016). The pervasive nature of social media and 24‐hour news cycles can amplify the impact of these stressors, as adolescents are constantly exposed to distressing images and narratives (Neria and Sullivan 2011). Moreover, the polarization of political discourse and the spread of misinformation can contribute to feelings of uncertainty and mistrust, further exacerbating anxiety symptoms (Strasser, Sumner, and Meyer 2022).

In addition to climate change and political instability, the COVID‐19 pandemic has negatively affected youth mental health. A review by the United States Surgeon General, identified several factors contributing to increased anxiety among young people during the pandemic, including having a frontline family member, living in an urban area with more severe outbreaks, disruptions in daily routines, and experiencing housing or food instability (Office of the Surgeon General 2021). Certain groups, such as LGBTQ+, those with disabilities, and individuals from lower socioeconomic backgrounds, faced additional challenges due to a lack of support resources and increased risk of negative mental health outcomes disproportionately impacting Black and Hispanic adolescents (Brooks et al. 2022).

As the long‐term consequences of climate change, political instability, and the COVID‐19 pandemic continue to unfold, it is crucial to prioritize the mental health needs of adolescents. Empowering adolescents with the tools and resources to build resilience, engage in activism, and foster a sense of community may help mitigate the psychological impact of these stressors (Sanson, Van Hoorn, and Burke 2019). Furthermore, teens can be aided by parents or close friends, helping them to be mindful of their media intake while supporting the information they process. Alongside these measures, teens can practice healthy self‐care habits and seek professional help. It is necessary to understand the mental impact of political and environmental change on the youth and to create safe spaces for children and adolescents to address their fears and concerns.

## 3 Discussion

The rise in adolescent anxiety can be attributed to numerous factors. We identified six current trends that contribute to anxiety among youth, including biological aspects, digital technology and social media, nuclear family dynamics, academia, extracurriculars, and political and environmental uncertainty. While other contributors exist, these variables were found to be consistently influential, especially diving into the specific susceptibilities of adolescents. Gen Z adolescents are uniquely vulnerable when compared to other generations due to their exposure and consumption of knowledge via social media and increased stressors from other aspects of life. We found that factors discussed in this review encompassed unique challenges that Gen Z adolescents face compared to other age groups, potentially explaining this difference in anxiety and other mental health disease occurrence. Anxiety has a four‐fold greater prevalence in Gen Z than the Baby Boomer generation and about two‐fold larger than Gen X (Grelle et al. 2023). These numbers are similar for other mental illnesses faced by Gen Z, including somatization disorder and major depressive disorder, underscoring the complexity of this topic. Understanding the origins of the current adolescent mental health crisis will allow the identification of potential treatment and prevention options.

Addressing adolescent anxiety requires navigating the complex intersection of contributing factors, each of which offers unique opportunities for intervention. The approach to adolescent anxiety requires a personalized treatment plan, as individual anxiety is likely to stem from only a few of the described factors. Interestingly, while there are validated and approved therapies for the treatment of adolescent anxiety and depression, there are no established guidelines for prevention (Walter et al. 2020). Both genetics and environment play a significant role in the development of anxiety, with certain genetic variants making an individual more susceptible to environmental insults (Ask et al. 2021; Gross and Hen 2004; Walter et al. 2023). Therefore, it is prudent to consider biological influences but understand that manipulation of biological variables may not always be a first‐line option, as genetic testing is expensive, and identification of certain genetic markers may not change the treatment plan. As such, mental health professionals, including psychiatric nurse practitioners, should consider modifiable risk factors as points of intervention.

As adolescent anxiety does not exist in isolation, it is critical to involve caregivers in the treatment process. Parenting styles can have a large impact on anxiety symptoms in adolescents (Romero‐Acosta et al. 2021). Supporting healthy parenting and encouraging healthy communication between the adolescent and their caregiver can provide emotional security and mitigate anxiety. Overuse of digital technology and social media, which is heavily correlated with increased rates of anxiety among the youth, is another area that may benefit from family intervention (Hamatani et al. 2022). Psychiatric nurse practitioners may consider counseling caregivers on limiting screen time and can assist caregivers in broaching the topic with the adolescent. Conversely, practitioners may choose to directly counsel the patient to avoid a parent–child conflict that could worsen anxiety symptoms in the adolescent. An alternative to screen time is extracurricular involvement, as it has been postulated that extracurriculars promote social interactions while decreasing exposure to social media and digital technology. Therefore, mental healthcare providers could consider educating families on the importance of engagement in extracurricular activities, while also advocating for schools and community centers to create and promote these resources.

There are some factors, such as the environmental crisis and global uncertainty, which induce anxiety in many adolescents yet fall outside of the control of the family. These feelings can be partially assuaged through education, as studies suggest that political and ideological education is linked to the reduction of anxiety symptoms in students (Zhang and Liu 2023). This finding also stresses the importance of not overlooking this component of adolescent anxiety, since teens are exposed to global political content daily through the use of social media. To combat false information and ease anxiety, schools may consider teaching students how to interpret political and scientific discordance. This can help manage anxiety if done in a benevolent and unbiased manner. Applying this idea to address the anxiety associated with global uncertainty is cumbersome but offers the potential for proactive, large‐scale prevention instead of reactive symptom management.

Additional ways that schools could assist in easing students’ anxiety is through various wellness initiatives. An important step is normalizing the conversation surrounding mental health and asking the students what they need to feel supported. Furthermore, schools could consider creating safe spaces where students can express their feelings, whether individually or in a group with their peers. Encouraging open communication with teachers, counselors, and parents can help identify significant concerns that may benefit from treatment. School‐based interventions for adolescents with anxiety have been explored extensively in the literature and focus primarily on providing cognitive behavioral therapy (Neil and Christensen 2009). However, while the general findings are promising regarding the utility of reducing anxiety symptoms, a recent 2020 meta‐analysis found that anxiety symptoms are only reduced transiently post‐intervention, and that current interventions implemented by schools are not supported by the existing evidence (Gee et al. 2020).

Early detection of these mental health issues and referral for evaluation and treatment by a mental healthcare provider like a psychiatric nurse practitioner can significantly improve youth mental health and prevent the development of more serious health problems. Additionally, healthcare providers can play a valuable role in fostering self‐esteem, which is an important foundation for mental well‐being (Fernandes, Newton, and Essau 2022). Therapeutic treatments that emphasize self‐esteem development may offer positive outcomes (Henriksen et al. 2017).

Exposure to nature has been proposed as a potential mechanism by which anxiety may be reduced. Several recent studies and literature reviews demonstrate that nature exposure can have a significant protective role on mental health (Browning et al. 2023; Jimenez et al. 2021; Kotera, Richardson, and Sheffield 2022; Li et al. 2018; Moll et al. 2022). An extensive narrative review conducted in 2021 evaluating the effects of nature exposure on a myriad of health outcomes found that increased nature exposure and access to greenspace leads to consistent improvements in physical and mental health (Jimenez et al. 2021). Similarly, a study evaluating the role of virtual nature simulation on anxiety in college studies found that anxious apprehension (worry) was significantly reduced in the sample of 40 students who participated in the study, and that reduction in anxiety and anxious arousal (panic) approached significance (Browning et al. 2023). These findings illuminate another potential therapeutic direction for adolescents with anxiety. Promoting time spent outdoors, whether it be through structured events, such as sports, or unstructured activities, could provide a potential outlet for anxiety that much of the youth can access. Further research needs to be conducted into the specific timeframe that adolescents should spend outdoors to gain this benefit.

Psychiatric mental health nurse practitioners are important providers of mental health services to adolescents and are heavily involved in the diagnosis and management of anxiety (Yang, Idzik, and Evans 2021). Psychiatric nurses play a significant role in mental health care, with the number of prescribing visits substantially increasing from 2011 to 2019 (Cai et al. 2022). Understanding the emerging root causes of anxiety in the Gen Z population will allow nurses to account for these factors during the assessment phase of treatment (Sampaio et al. 2021). Additionally, this information can improve treatment by enhancing the therapeutic alliance between the patient and the care team (Hartley et al. 2020).

## 4 Conclusions

Adolescent anxiety is influenced by a multitude of factors that encompass biological, environmental, and social domains. Understanding these factors can aid in identifying at‐risk youth and developing effective interventions and treatment strategies, thereby facilitating the care provided by psychiatric nurses.

## Author Contributions

Thea L. Anderson and Rasa Valiauga conceived the idea for the review and led the project. Thea L. Anderson, Rasa Valiauga, Christian Tallo, Catriona Blythe Hong, Sham Manoranjithan, Catherine Domingo, Manasvi Paudel, Ana Untaroiu, and Samantha Barr conducted the literature search and data analysis. Kate Goldhaber contributed to the design of the review framework and provided critical feedback on the manuscript. All authors contributed to writing and revising the manuscript, approved the final version, and agree to be accountable for all aspects of the work.

## Ethics Statement

The authors have nothing to report.

## Conflicts of Interest

The authors declare no conflicts of interest.

## Data Availability

The authors have nothing to report.
